# 

**Published:** 1890-03-15

**Authors:** 


### NEW OFFICES IN THE STRAND.

The Hospital *is now published at* **140. Strand, W.C.** *It has been found desirable to establish* The Hospital *in this central and convenient locality, owing to the rapid extension of the business of this paper of late.*

### NOTICE TO CORRESPONDENTS.

All MS., Letters, Books for Review, and other matters intended for the Editor should be addressed The Editor, The Lodge, Porchester Square, London, W.

**Notice to Advertisers and Subscribers.**

All Advertisements, Orders for Copies of The Hospital, and Business Communications should be addressed to The Publisher, *not to the Editor*, The Hospital, 140, Strand, W.C.

**Bound Volumes of "The Hospital."**

Vol. VI., containing full page portraits of T.R.H. the Prince and Princess of Wales, and Miss Florence Nightingale, is now ready, 4s. post free. Cases for binding may be had of the Publisher, at 1s. 6d. each.

**To Residents, Secretaries, and Matrons.**

The Editor will be glad to have the Regulations and each Report as issued by Asylums, Hospitals, Convalescent and Nursing Institutions, as well as those of other Charities, and an early copy in duplicate of all Appeals and Festival Notices, etc., addressed to *The Editor, The Lodge, Porchester Square, London, W.* Any superintendent, secretary, matron, or other chief official who regularly sends such information may be registered, on application to the Publisher, to receive free of cost a cover for binding The Hospital in half-yearly volumes.

### Scraps and Gleanings.

Surbiton Cottage Hospital treated 142 patients last year.

The Edinburgh Sanitary Protection Association is increasing its members.

Mr. Bullen and Mr. Frames, who are leaving Banstead Asylum, are going to the Cape.

Warrington Infirmary treated 198 patients last year at a total expenditure of £1,370.

Influenza is very prevalent in the North of Scotland, 30 deaths having occurred in Wick alone.

Dr. Alice Bennett has been elected president of the Montgomery County Medical Society, U.S.

Halifax is not only in need of a new infirmary, but also of a new fever hospital, which Dr. Arnley says should be free.

The Hahnemann Convalescent Home at Bournemouth treated 158 patients last year, the largest number on record.

Kent County Ophthalmic Hospital issues a good report. The High Sheriff of Kent presided at the annual meeting.

The London Hospital is to be enlarged: a new theatre, chapel, and receiving-room are to be erected in front of the present building.

Mr. Francis H. Edgeworth, M.B., B.Sc., has been appointed one of the hon. physicians to the Bristol Hospital for Sick Children and Women.

Mr. H. A. Brassey presided at the eleventh annual dinner in aid of the East London Hospital for Children, and made an eloquent appeal for funds.

The Clergy Orphan Schools Committee are appealing for funds, by circulating the speech of Lord Cranbrook, made at their annual meeting in February.

The epidemic some time ago raised the price of quinine. It is now reported from Rotherham that the demand for medicine bottles is unusually acute,

Three lunatics came before the Thames Police Court last Saturday. Dr. Houchin, who was in attendance, said he believed insanity was greatly on the increase.

The late Mr. Michael Beveridge, who was Provost of Kirkcaldy, has bequeathed to its inhabitants the sum of £50,000 for the purchase of a public park and library.

The house-staff of the Eastern Hospital at Brooklyn have resigned because they say they have too many cold meals, and the bed linen leaves much to be desired.

Madame Albani has promised to sing, in June, in aid of the National Society for the Prevention of Cruelty to Children. The Princess of Wales will be present at the concert.

Morley House Convalescent Home proposes to augment its funds by a great parade of friendly societies before the Lord Mayor and Sheriffs in the Royal Albert Hall on May 10.

Influenza in Manchester and district, though late in appearing, is now prevailing to a considerabe extent, not so much in the city itself as in the surrounding towns and outlying districts.

The *Dublin Gazette* announces that his Excellency the Lord Lieutenant has appointed Dr. Edward Courtenay to be an inspector of lunatic asylums in Ireland, in the vacancy created by the retirement of Dr. John Nugent.

Vienna has no fewer than five women who are all aged over a hundred. The one who has the strongest claim to the pension of £24 granted by the municipality to the late Magdalena Panza, is said to be upwards of 115 years of age.

The treasurer of the Royal Westminster Ophthalmic Hospital has recently acknowledged the gift of £50 from an anonymous donor, signing himself "Grateful," in recognition of a successful operation performed on his father upwards of fifty years ago, by the late Dr. Guthrie.

The opposition to Sir Robert Rawlinson's scheme for the disposal of London sewage is extending in Essex, and the Colchester Corporation, who hold by royal charter certain rights over the oyster fisheries of the Colne, resolved yesterday to present a memorial to the London County Council on the subject.

The death of Mr. Alderman Stone, who, since his mayoralty in 1875, has taken little or no part in Corporation matters, places at the disposal of the Governors of St. Thomas's Hospital the desirable post of treasurer, to which is attached a charming residence opposite the Houses of Parliament, with certain other advantages.

A sharp controversy on "Cremation" has been called forth in the French capital since the Cardinal Archbishop of Paris issued his circular in condemnation of the practice of burning the dead. Various persons—lay and clerical—have given their opinions on the subject, and among the opponents of "Cremation" is M. Ernest Renan.

It appears from the twenty-fifth annual report of the Peabody Trust that the birth-rate in their dwellings is 8·72 per 1,000 above the rest of London, and the death-rate very nearly 1 per 1,000 below, while the mortality of infants is not much more than half. The trust has spent over a million and a quarter sterling in housing 20,274 persons.

Johannesburg is a nice place for teetotallers. Dr. Matthews, who recommended that the Sanitary Board of that town should take the water supply into their own hands and bring water from the Vaal, a distance of thirty miles, declares that in every 75,000 gallons supplied by the company there is a ton of solid matter, to which fact he attributed the abnormally high death-rate from typhoid fever.

Mr. Gladstone will open the new Medical College at Guy's Hospital on Wednesday, the 26th inst. The college consists of dining-hall, reading-rooms, and gymnasium, and accommodation for the junior members of the staff and fifty resident medical students. Dr. Perry (Dean of the Medical School) will be the first warden of the college. A subway has been constructed between the college and the hospital.

